# Community stigma and discrimination against the incidence of HIV and AIDS

**DOI:** 10.25122/jml-2023-0171

**Published:** 2023-09

**Authors:** Andi Asrina, Muhammad Ikhtiar, Fairus Prihatin Idris, Arlin Adam, Andi Alim

**Affiliations:** 1 Faculty of Public Health, Universitas Muslim Indonesia, Makassar, Indonesia; 2 Faculty of Public Health, Universitas Pejuang Republik Indonesia, Makassar, Indonesia

**Keywords:** HIV/AIDS, stigma, discrimination

## Abstract

The problem of human immunodeficiency virus (HIV) /acquired immunodeficiency syndrome (AIDS) is increasingly complex, including not only health-related concerns problems but also rampant stigma and discrimination, further exacerbating the health and social conditions of the affected individuals. This study aimed to examine the triggers of stigma and discrimination among individuals with HIV/AIDS in Wakatobi, Southeast Sulawesi. We employed a quasi-qualitative design with a case study approach involving data reduction, presentation, and drawing conclusions. Triggers of stigma and discrimination experienced by individuals living with HIV/AIDS encompassed a range of factors. Stigma was rooted in various causes, including fear, perceptions of unattractiveness, anxiety, associations with the disease, and lack of confidentiality. Discrimination, on the other hand, was caused by disappointment, feelings of insecurity, diminished self-esteem, and factors like competition and exploitation. The forms of stigma for people living with HIV/AIDS were public stigma, self-stigma, verbal discrimination, and avoidance. Meanwhile, the impact of stigma and discrimination on individuals living with HIV and AIDS is substantial. They encounter difficulties in finding help, restoring their lives, and discrimination. Stigmatizing attitudes and discriminatory acts of people living with HIV/AIDS worsen the quality of life of people living with HIV/AIDS, which can potentially cause new infections. Therefore, the government should undertake extensive educational initiatives regarding HIV and AIDS. By enhancing public knowledge and awareness, society can work towards eradicating stigma and discrimination from social interactions.

## INTRODUCTION

The 2030 global target for the prevention of human immunodeficiency virus (HIV) and acquired immunodeficiency syndrome (AIDS) aims for zero new HIV infections, zero AIDS-related deaths, and zero stigmatization and discrimination [1]. The goal of achieving zero stigma and discrimination targets people living with HIV/AIDS (PLWHA) who are likely to experience obstacles due to a lack of understanding in society, accompanied by prejudiced attitudes [2]. HIV is, by definition, a virus that attacks the human immune system and weakens the body's ability to fight all types of disease. AIDS is a collection of disease symptoms caused by a decrease in the immune system resulting from HIV infection [3]. AIDS is often referred to as a disease of deviant behavior because the factors that trigger this event were previously only found in at-risk groups such as commercial sex workers, individuals with diverse sexual orientations, injecting drug users, and individuals who engaged in risky sexual behaviors [4].

Currently, HIV and AIDS have become a generalized epidemic, affecting individuals across diverse demographics, including individuals from various backgrounds and children, without regard to age, gender, occupation, ethnicity, or nationality, highlighting the sociological and public health significance of these diseases, influencing all societal strata [5]. According to the Report on HIV, AIDS, and Sexually Transmitted Diseases in Indonesia up to September 2022, 338,760 people were living with HIV (PLHIV), with 140,024 AIDS cases by June 2022, bringing the total HIV and AIDS cases to 478,784 by June 2022 [6].

Southeast Sulawesi has witnessed an increase in the findings of new HIV/AIDS cases every year. According to data, the number of HIV/AIDS cases in Southeast Sulawesi Province between 2012 and 2017 has increased. According to the Report on the Development of HIV, AIDS, and Sexually Transmitted Diseases, for the first quarter of 2021 in Southeast Sulawesi, 16 new HIV cases were detected among 3,795 individuals tested. Of 5,678 people tested for HIV during April-June 2022, 95 cases of HIV/AIDS were identified [7].

These figures only represent individuals who take the test voluntarily. Furhtermore, Wakatobi District has also experienced an increase in the number of new cases. Data from the Wakatobi Regional AIDS Commission indicates 13 new cases of HIV in 2021 and 16 new cases in 2022 [8]. In reality, the actual number of cases is significantly higher in all regions due to the underreported cases of HIV/AIDS, driven by stigma and discrimination against those affected. Subsequently, the population at risk tends to withdraw from taking the test. In effect, new infections occur in the form of silent epidemics. Stigma and discrimination often occur in families, communities, schools, places of worship, workplaces, and legal and health services.

The process of silent transmission can be facilitated by various factors within at-risk populations. For example, individuals engaged in long-term overseas work may face an increased risk of engaging in risky sexual behavior, which can contribute to silent transmission. People living with HIV/AIDS consist of various age groups, genders, groups, and statuses. Both people who suspect they may be infected with sexually transmitted diseases and those already living with HIV usually withdraw from themselves and fear stigma and discrimination, making it challenging to develop prevention, care, and treatment practices.

Numerous studies, including research by Mahamboro *et al*., found that individuals with HIV/AIDS face external stigma in healthcare facilities, communities, and families, leading to discriminatory attitudes and behaviors [9]. They also experience anticipatory stigma due to other PLWHA's experiences of stigma and discrimination. Individual moral judgments about HIV status and negative self-assessment are determinants of perceived stigma. Similar research was also conducted by Fauk *et al*., suggesting that stigma and discrimination against PLHIV still exist in the family, community, and health services. This is reflected in negative labeling, segregation of personal belongings, avoidance, treatment refusal, and rejection of PLWHA by healthcare providers, families, and community members. Some healthcare providers even admit that they are also involved in stigmatizing and discriminating PLHIV [10].

Fauk *et al*. found that participants experienced stigma and discrimination in various places, including the family, community, and healthcare settings. Factors such as lack of knowledge about HIV, fear of contracting HIV, and social and moral perceptions about HIV and PLHIV are considered drivers of stigma and discrimination [11]. Follow-up research conducted by Fauk *et al*. found that HIV causes negative psychological consequences for WLHIV, such as stress, fear, worry, anxiety, and depression, as well as social impacts, including stigma, discrimination, and family separation [12]. In addition, Fauk stated that the impact of HIV on women living with HIV (WLHIV) and men living with HIV (MLHIV) and their families included (i) stigmatization and discrimination, (ii) psychological impact, (iii) family conflict and separation, and (iv) physical impact [13].

Despite facing psychological challenges, stigma, and discrimination, WLHIV demonstrated resilience in adopting effective coping strategies and seeking support to educate themselves and their communities. These findings underscore the importance of policies and practices that assist WLHIV in addressing HIV-related psychological and social challenges [14].

Many studies have reported that stigma and discrimination occur within one's family, such as siblings, relatives, and even parents. This often results from the fear of HIV transmission and remains a persistent global challenge in reducing infection rates [11, 15-17]. The fear of contracting HIV occurs from a lack of knowledge about HIV and is a driving factor for stigma and discrimination against people living with HIV [14, 18, 19]. Responses resulting from immoral, dirty, embarrassing, and inappropriate behavior further increase the stigma and discrimination against PLHIV [9, 20, 21].

Various studies on stigma and discrimination against PLWHA are generally based on a positivistic paradigm that views events of stigmatization and discrimination as objective facts. This study rests on the constructivism paradigm by focusing on the meanings constructed by the community that shape their knowledge and actions towards PLHIV. The epistemology of constructivism research uses the social construction theory by Peter L. Berger and Thomas Luckmann as analytical tools. In explaining the constructivist paradigm, social reality is a social construction individuals create through social interaction. Knowledge is seen as a picture that is formed from objective reality in itself. The individual becomes a determinant in the social world constructed based on his will. Individuals are not victims of social facts but of creative production and reproduction media in constructing their social world [22-25].

Individuals continuously create a reality that is subjectively owned and shared and forms standards and values recognized by society [26-28]. This intricate interplay involves individuals creating society and society creating individuals through externalization, objectivation, and internalization [29]. This study aimed to analyze the causes, forms, and impacts of stigmatization and discrimination of people living with HIV/AIDS as a product of dialectical social interactions.

## MATERIAL AND METHODS

### Epistemological foundation and research approach

This study adopts the perspective of Peter L. Berger and Luckmann's Social Construction Theory, which views social reality as relative and created by individuals. This paradigm determined the choice of research methods, data collection, data analysis, and interpretation. Consequently, we used a qualitative approach because it emphasized the reality created by individuals, using a case study method to explore the phenomena of stigma and discrimination against people living with HIV and AIDS due to a social construction process.

### Study location and participants

The research was conducted in the Wakatobi District due to the increasing cases of HIV/AIDS in the region and the ongoing mentoring and monitoring programs for suspected and confirmed HIV cases. This environment facilitated access to relevant informants and information. The informants for this study were HIV and AIDS personnel at the Wakatobi Health Office as key informants, representatives of the Regional AIDS Commission as supporting informants, and key informants. The selection of these informants was purposeful, targeting individuals with strong communication skills and a willingness to participate in interviews. The selection of informants was based on consideration of their direct involvement in the social world, which reflects the reality of stigmatization and discrimination of people living with HIV/AIDS as the basic assumptions of the constructivism paradigm, which forms the basis of this research.

### Data collection

This research used various tools for data collection, including interview guides, observation sheets, field notes, cameras, recorders, and supplementary materials. Data was collected through observation, in-depth interviews, and documentation. Due to the constructivist paradigm guiding this research, the researcher's values, ethics, and morals played a role in interpreting the data. This recognition is essential because it acknowledges that the meaning of data is influenced by the researcher's subjective interaction with individuals who have experienced stigma and discrimination related to HIV/AIDS. There are three components in qualitative research called social situations: places, actors, and activities. Observations were made to observe informants' activities on the phenomena being investigated, either directly or indirectly. Meanwhile, in-depth interviews explored invisible phenomena by tracing information from all informants regarding stigma and discrimination. Documentation relevant to the review is a source triangulation approach to ensure the validity of the qualitative findings.

### Data analysis

The qualitative data analysis in this study followed the Miles and Huberman model, guided by a constructivist approach. This model outlines several key stages of analysis. First, data reduction involved categorizing and selecting relevant data to compile the collected information. Next, data were displayed to facilitate drawing conclusions and informing subsequent actions. Following this, conclusions were drawn and verified. Ultimately, the findings were presented in a narrative form. Throughout these stages, constructivist principles were applied, involving a reasoning process informed by the researcher's creative insights. This approach was essential for comprehensively explaining the findings and constructing a nuanced understanding of the research.

## RESULTS

As shown in Table 1, most informants were between the ages of 23 and 54, with varying education and occupations, and HIV positive. Despite having higher levels of education, some informants lacked knowledge about HIV, and their occupations did not necessarily involve efforts to prevent HIV transmission or promote openness about their HIV status, which can lead to stigma and discrimination.

**Table 1:** Informants’ characteristics

No	Informant Initials	Age	Gender	Education	Work	Information
1	Ni	35 years	Woman	3-Year Diploma	Civil Servant	Key Informants
2	Kj	40 years	Man	Senior High School	Honorary	Common Informant
3	Ra	37 years	Woman	Senior High School	Housewife	Common Informant
4	Ai	23 years	Man	Student	-	Common Informant
5	Zn	41 years	Man	Senior High School	Private	Common Informant
6	Wu	28 years	Woman	3-Year Diploma	-	Common Informant
7	Tn	54 years	Man	Senior High School	Private	Supporting Informants

### Causes of stigma and discrimination against HIV and AIDS

The study revealed that stigma and discrimination can arise due to fear of HIV and AIDS. This fear is experienced in the same way, both in the family environment, work, social environment, or health services in general, as expressed by the following informants:

*“If someone is exposed to (HIV)? That is contagious, it is because they've engaged in risky behavior, and that's why they get infected like that”* (Mr., 54 years)

These perspectives depict HIV as a virus that can move around easily and even be transmitted through ordinary social relationships. This point of view grows and develops due to the internalization process received in social interaction, which generally has a wrong understanding of how HIV is transmitted. This means that individuals create a process of social construction, which is understood collectively and then develops into general knowledge.

Misunderstandings that have been objectivated by society are used as a basis for externalization in the form of stigmatizing and discriminating against those affected. Because of that, people with HIV also experience construction in the form of fear due to their HIV-positive status. The implication is that individuals affected must be forced to lie and cover up the disease, as revealed by the following informants:

*"I was diagnosed HIV positive in 2019; of course, I was shocked...what if someone finds out later, but now I have been taking medication for 3 years...If someone asks me what's wrong, I answer that I have TB, so I have to take medicine”* (Kj, 40 years)

*“Initially, my wife was surprised. Now, others know too, and they avoid us, gossip about us, and refuse to interact. No one wants to shake hands”* (Kj, 40 years)

The behavior of people with HIV and their families who cover their HIV status is a way of dealing with society's response, which tends not to provide reasonable social acceptance for people living with HIV. For those affected, the treatment of society in the form of being shunned, blamed, and not even wanting to shake hands is much more painful than the physical suffering caused by the disease.

In line with what was experienced by the previous informant, another informant also revealed:

*"As soon as they know someone is HIV positive, they avoid walking in front of the house of those known to have HIV"* (Ra, 37 years).

According to an informant, the community's strong response to people living with HIV is practiced by avoiding walking in front of the house, let alone meeting people with HIV, because they consider it a disgrace. This means that individuals affected are already isolated in the interaction process because society perceives them as violators of social norms that should be avoided. These individuals experience difficulties accessing services, especially medical services that they must undergo routinely. Furthermore, they do not adhere to medication due to the difficulty building interactions with the social environment.

### Forms of stigma and discrimination against people with HIV/AIDS

Perceptions that arise from the family and social environment of people living with HIV are generally negative, as stated by the following informants:

*"We, the sufferers, are thought to be sinners because we are infected through commercial sex, so people often tell negative stories when they are together. We finally think we have no value in the eyes of society”* (Ni, 35 years old)

The label of a sinner is the impression captured by the person based on the community's response to him. Generally, people understand that HIV is only transmitted through sexual intercourse with multiple partners. Because of that, society uses its moral basis in treating people living with HIV. The construction of this knowledge comes from the religiosity of society, which is the principle of developing daily behavior.

Another form of stigma accepted by individuals with HIV/AIDS is that society views them as a dangerous transmission source. Because of that, there are no social spaces for them to live, including their families. This feeling was experienced by the informant below:

*"The general opinion is that people with HIV are the ones who transmit HIV. That's why people don't give people the opportunity to socialize. What's worse, the family shares the same opinion so that the sufferer is forced not to be accepted by the family”* (Zn, 41 years old)

The partial understanding of the public regarding the mode of transmission that only sees sexuality has perpetuated a dangerous response for the future lives of individuals living with HIV. Society closes itself, subsequently influencing the attitudes of families caring for their HIV-positive members. This social process clearly shows the existence of a reality created by socially accepted individuals and then socialized in everyday life to form norms in the form of rejection for people living with HIV/AIDS.

### The impact of stigma and discrimination on people with HIV/AIDS

The occurrence of stigma and discrimination certainly has an impact on PLWHA, and some are afraid to seek treatment:

*“As I said, if HIV is known, they will be shunned, talked about carelessly, blamed, so they don't dare to get tested”* (Al, 23 years)

The discriminatory treatment resulting from negative social stigma can lead individuals to postpone undergoing HIV testing. This delay can have several consequences, including an increased risk of individuals engaging in risky behavior and developing preventable opportunistic infections that could have been detected early through testing. Furthermore, such delays contribute to the persistence of the HIV iceberg phenomenon, as individuals fear public avoidance upon disclosing their HIV-positive status. Another impact found the neglect of the affected individual's quality of life due to their reduced life expectancy, as highlighted by the following informant:

*"It's useless to take medicine all the time because our families don't think we're humans anymore, and it seems that society doesn't want to hang out anymore...Why else live when it's like this”* (Wu, 28 years)

The absence of reasonable life opportunities in society makes people despair; some even experience symptoms of mental disorders. Consequently, people with HIV/AIDS may neglect medication regularly and fail to engage in other healthy behaviors. This neglect has a detrimental impact on the quality of life of those with HIV/AIDS, leading to a decline in health until they face imminent death. Even though ideally, when individuals with HIV maintain healthy behaviors, they can live productive lives similar to those without HIV.

This information was corroborated by local health officials as follows:

*"The latter was a case of a woman who was depressed because she could not accept her HIV status; in the end, the collaboration was treated by a psychiatrist... Indeed, some people here generally don't know what HIV and AIDS are, the causes of which are widely known as infectious diseases due to free sexual behavior and injecting drugs. So we need information, that’s why we are educating school children... It is really necessary to provide information widely because this disease occurs in all places... Stigma and discrimination exist because of people's ignorance”* (Ni, 35 years)

## DISCUSSION

### Causes of stigma and discrimination against individuals living with HIV/AIDS

Stigma and discrimination against people living with HIV/AIDS were caused by misunderstanding, excessive fear, social disgrace, and the notion that people with HIV and AIDS violate religious and social norms. The lack of public knowledge about the pattern of HIV transmission can lead to stigma and discrimination. This study aligns with the findings of Fauk *et al*., who identified erroneous knowledge as a driver of stigma. This erroneous knowledge is formed through the process of daily social interaction, which becomes the basis for the process of externalization, as explained by Peter Berger regarding the process of social construction, which involves the process of internalization, objectivation, and externalization [11].

Wrong knowledge creates excessive fear, which causes stigmatization and acts of discrimination. Fauk *et al*. stated that fearful attitudes can lead to stigma and discrimination against people living with HIV and AIDS by focusing on the implications of the objectivation process in Peter Berger's social construction theory. Within the Wakatobi community, the moral perspective of HIV/AIDS has undergone a process of institutionalization (objectivation) and has become the basis for external actions. The typology of the Wakatobi religiosity community also influences this basis of morality. Fear is the main cause of stigma and discrimination against people living with HIV and AIDS. The community thinks HIV and AIDS are only experienced by at-risk people, such as drug users and multiple sexual partners, and can be transmitted through touch. Fear of infection is also a source of discrimination, even by families and health workers [30, 31].

Fear is the initial cause of stigma in people with HIV and AIDS. These individuals often perceive HIV as an incurable, life-threatening infectious disease, leading them to believe they must isolate themselves to prevent transmission. This feeling of fear is rooted in a lack of understanding about HIV, causing those affected to conceal their status due to insufficient information and lack of self-confidence. This leads to social isolation, dissemination of HIV status, and rejection, making it difficult to detect the number of HIV cases and hindering access to and adherence to HIV prevention and treatment efforts [11, 32-34].

AIDS stigma occurs due to the belief that AIDS is a disgrace that causes anxiety and discomfort. Goffman stated that this anxiety is a disability due to the HIV and AIDS virus, so sufferers feel insecure in interacting and dealing with other people, inhibit social relations, have negative views of themselves, and feel rejected by the surrounding environment [35-38].

This study revealed that individuals living with HIV/AIDS often experience discomfort, anxiety, and a sense of shame associated with their condition. These feelings hinder their interactions with others. Consequently, the social implication is that people with HIV and AIDS tend to isolate themselves, withdraw from social activities, and believe they do not deserve to be part of their families or communities due to their perceived differences arising from their HIV and AIDS status.

Individuals living with HIV and AIDS are aware that despite the actual cause of their infection not being related to unprotected sex, it is challenging for society to shift the prevailing belief that HIV is primarily transmitted through sexual promiscuity. Misconceptions and stereotypes sometimes associate HIV and AIDS with what some consider 'immoral' behavior, including activities associated with groups such as commercial sex workers, LGBTQ+ individuals, and drug users. Religious thinking that considers free sex and multiple partners to be unlawful and a sin also triggers negative evaluations from society toward people with HIV [39, 40].

Based on an understanding of morality, people with HIV and AIDS are also labeled violators of social and religious norms. Social norms relate to rules of sexual behavior, which are only accepted if a marriage bond exists, and religious norms prohibit sex outside of marriage. Society considers that people living with HIV and AIDS do not comply with this norm, so they get the disease. Therefore, they are considered violators of norms and often face societal avoidance to avoid ridicule. This social process reflects the construction of reality by society based on the existing social order.

Social construction is formed through internalization, in which individuals interpret and understand according to standard norms. Then, the objectivation process produces externalization actions. In this study, stigma and discrimination against people living with HIV and AIDS result from social constructions based on people's perspectives, interpretations, and understanding of these conditions. After contracting HIV and AIDS, a person will adjust as a person with HIV and face the stigma and discrimination that arise from society.

### Forms of stigma and discrimination against people with HIV and AIDS

The research found two forms of stigma and discrimination against people living with HIV and AIDS: public stigma and self-stigma. Forms of discrimination include verbal discrimination and avoidance, as shown in Table 2.

**Table 2:** Forms of stigma and discrimination against people living with HIV and AIDS

Acquired forms of stigma	
**Theme**	**Sub-theme**
Public Stigma	• Labeling • Stereotypes • Exclusion • Loss of status • Minimal social support from family and community
Self-Stigma	• Powerlessness • Low self-efficacy • Withdrawal • Emotional responses such as fear, anger, disappointment, shame • Hopelessness • Loss of appreciation/self-esteem
**Forms of Discrimination**	
Verbal expression Avoidance	Avoidance/shunning

Public stigma occurred from the family and social environment. Overreaction to the words HIV and AIDS caused public stigma. Society often associates HIV with sexual behavior outside of marriage and drug use so that someone infected with HIV can be labeled negatively. People living with HIV face social stigma because it is considered a disease that is scary, embarrassing, and a violation of social norms, which causes them to be ostracized and shunned [41, 42]. This stigma is an unfair prejudice often generalized to all people living with HIV due to the lack of information and understanding about HIV and AIDS and misinterpretation of the information received.

Self-stigma is another form of stigma in which people with HIV experience decreased self-esteem and self-confidence. In this study, self-stigma was often found in people living with HIV and AIDS after being declared HIV positive. They faced negative thoughts, distanced themselves from their families, felt hopeless, ashamed, angry, and withdrew from social circles. This self-stigma keeps their quality of life low, causing anxiety, depression, and even suicidal ideation [43].

The research results on discrimination revealed several forms, including verbal discrimination, avoidance, and exclusion. Verbal discrimination involves insulting words directed at people living with HIV and AIDS. This often happens due to a lack of information about HIV/AIDS, leading to demeaning words that isolate and devalue individuals with the condition.

Discrimination in the form of avoidance involves actively avoiding or staying away from people or groups that are disliked. Changes in the status of people living with HIV and AIDS result in changes in behavior in the surrounding environment. People with HIV and AIDS feel that once close people now act as if they do not know them. Previous good relationships become tenuous, and they do not want to be close to or touch and tend to stay away and avoid.

### The impact of stigma and discrimination on people with HIV and AIDS

Stigma and discrimination against people living with HIV and AIDS are not inherent but the result of a social process in which meanings and norms are built and maintained. Society co-creates stigmatization of HIV and AIDS through stereotypes, moral judgments, and negative assumptions about infected people. This stigma is a stereotyped activation of certain individuals or groups, which always has a negative impact, causing a decrease in self-esteem and poor self-efficacy in people affected [44, 45]. Social construction theory emphasizes the role of symbols, language, and narratives in shaping social reality. In the context of HIV and AIDS, the use of certain terms such as “AIDS sufferers” or other derogatory words can reinforce stigma and discrimination against people with HIV.

Community perceptions of symbols in the form of words influence patterns of social interaction with people living with HIV. If society generally avoids or isolates them, the stigma will get stronger and make sufferers feel alienated. This study is in line with the findings of Fauk *et al*., who found that stigma and discrimination against people with HIV and AIDS still exist within the family, community, and health services. This is reflected in negative labeling, separation of personal belongings, avoidance, resistance to treatment, and rejection by healthcare providers, families, and communities. Some healthcare providers even admit to being involved in stigmatizing and discriminating against people living with HIV and AIDS [10].

Institutions such as government agencies, health services, and workplaces can influence the reproduction of stigma and discrimination. Discriminatory practices against people living with HIV and AIDS can make them reluctant to seek support from health services, including HIV testing [11]. Research by Mahamboro *et al*. shows that informants experience external stigma in health facilities, communities, and families, expressed through discriminatory attitudes and behavior. They also experience anticipatory stigma because of their experiences of stigma and discrimination from other people with HIV and AIDS. Individuals' moral judgments regarding HIV status and negative self-assessments contribute to perceived stigma [9]. When individuals affected by HIV do not seek health services and support, it becomes challenging to determine their HIV status. This lack of awareness can contribute to the HIV iceberg phenomenon, wherein many undiagnosed cases exist. Consequently, this situation increases the risk of new HIV infections emerging within the population.

Stigma in healthcare facilities is a symptom of objectivation in social construction theory. People experiencing stigma increasingly feel unaccepted because credible health services show negative attitudes and perceptions. Feeling ignored, unacknowledged, and rejected by healthcare facilities can cause individuals to lose motivation to live a healthy lifestyle. This condition causes PLWHA to withdraw and become reluctant to socialize or utilize health services for their treatment. This discrimination impacts non-compliance with treatment and hinders the healthcare process for PLHIV [46].

Stigma and discrimination against HIV and AIDS also seriously impact the mental health of people with HIV/AIDS. Those who face discrimination and social rejection often experience heightened stress, anxiety, and depression, leading to hopelessness and a loss of motivation to prioritize their health. This finding is supported by Darak *et al*., which state that people who live in the same house as people with HIV who are not infected also experience mental health problems [47].

Moreover, stigma and discrimination can influence the attitudes of people with HIV/AIDS towards medical care and healthy behavior. Feelings of hopelessness and mental disorders can lead to reluctance to seek medical attention and neglect preventive measures essential for maintaining health. This research is in line with the results of Fauk *et al*., which shows that HIV causes negative psychological consequences among people with HIV, such as stress, fear, worry, anxiety, and depression, as well as social impacts, including stigma, discrimination, and family separation [12].

To overcome the negative impact of stigma and discrimination, which often lead individuals living with HIV and AIDS to neglect healthy behaviors, an approach rooted in social construction theory can help. Accurate education about HIV and AIDS, strong anti-stigma campaigns, positive social support, and reduction of internal stigma can help reduce the negative impact of stigma and discrimination. Thus, individuals with HIV/AIDS can feel more supported and motivated to take positive steps in maintaining their health.

Fauk *et al*. noted the psychological challenges, stigma, and discrimination faced by WLHIV. However, they demonstrate an extraordinary capacity to adopt effective strategies and support to protect and educate themselves and those around them. These findings highlight the need for policies and practices that help WLHIV better address HIV-related psychological and social challenges [14].

## CONCLUSION

The stigmatization and discrimination faced by individuals living with HIV and AIDS result from community construction. This process is primarily caused by societal fear, rooted in misconceptions about HIV events and based on interpretations of morality and religion. Consequently, individuals living with HIV are often unfairly labeled as disgraceful and violators of social and religious norms who have committed deviant behavior. The forms of stigma and discrimination are public stigma, self-stigma, and verbal avoidance. These harmful consequences include decisions to avoid HIV testing, stop using health services, ignore healthy behaviors, and develop mental disorders that worsen the quality of life for people with HIV/AIDS. It is recommended to involve key people such as health workers and individuals with HIV/AIDS in actively carrying out the process of social reconstruction. Health workers apply friendly behavior in providing health services, and individuals with HIV/AIDS dare to disclose their status and actively campaign for the social support they need.

## Data Availability

The data is available from the corresponding author upon reasonable request.

## Personal thanks

We extend our gratitude to the University of Muslim Indonesia for their support, the Wakatobi Regency Government in Southeast Sulawesi for granting us research access, and our valued participants for their willingness to engage in our study.
